# CMR characterization of atrioventricular inflow in constrictive pericarditis - findings of blood impact physiology

**DOI:** 10.1186/1532-429X-17-S1-P296

**Published:** 2015-02-03

**Authors:** George O Angheloiu, Geetha Rayarao, Mark Doyle, June A Yamrozik, Ronald B Williams, Robert W Biederman

**Affiliations:** 1 Cardiology, Penn Highlands, Dubois, PA, Dubois, PA, USA; 2 Allegheny General Hospital, Pittsburgh, PA, USA

## Background

With CMR septal bounce is seen with each cardiac beat while images are acquired during apnea, which leads to our hypothesis that septal bounce in constrictive pericarditis (CP) is a result of intrinsic cardiac phenomena, namely the impact between the atrioventricular inflow and the intervetricular septum.

## Methods

We compared SSFP four chamber view images (20 phases per cycle protocol) between 11 surgically-confirmed CP patients and 11 controls. We deconvoluted the mechanics of the septal bounce and correlated it with periods of the cardiac cycle. To demonstrate that the septal bounce is caused by the interaction between the tricuspid inflow and septum we constructed a parameter called the septal flow ratio equal with the ratio between the tricuspid inflow directed towards the interventricular septum and the total tricuspid inflow at peak rapid ventricular filling, as seen in the Figure. Assuming that the tricuspid inflow is perpendicular on the tricuspid valve annulus plane, the dark rectangles represent the inflow impacting the septum while the white rectangles represent the inflow impacting the right ventricular free wall, with their sum representing the total inflow. Left panels are 3 examples of CP while on the right are 3 normal patients.

**Figure 1:**
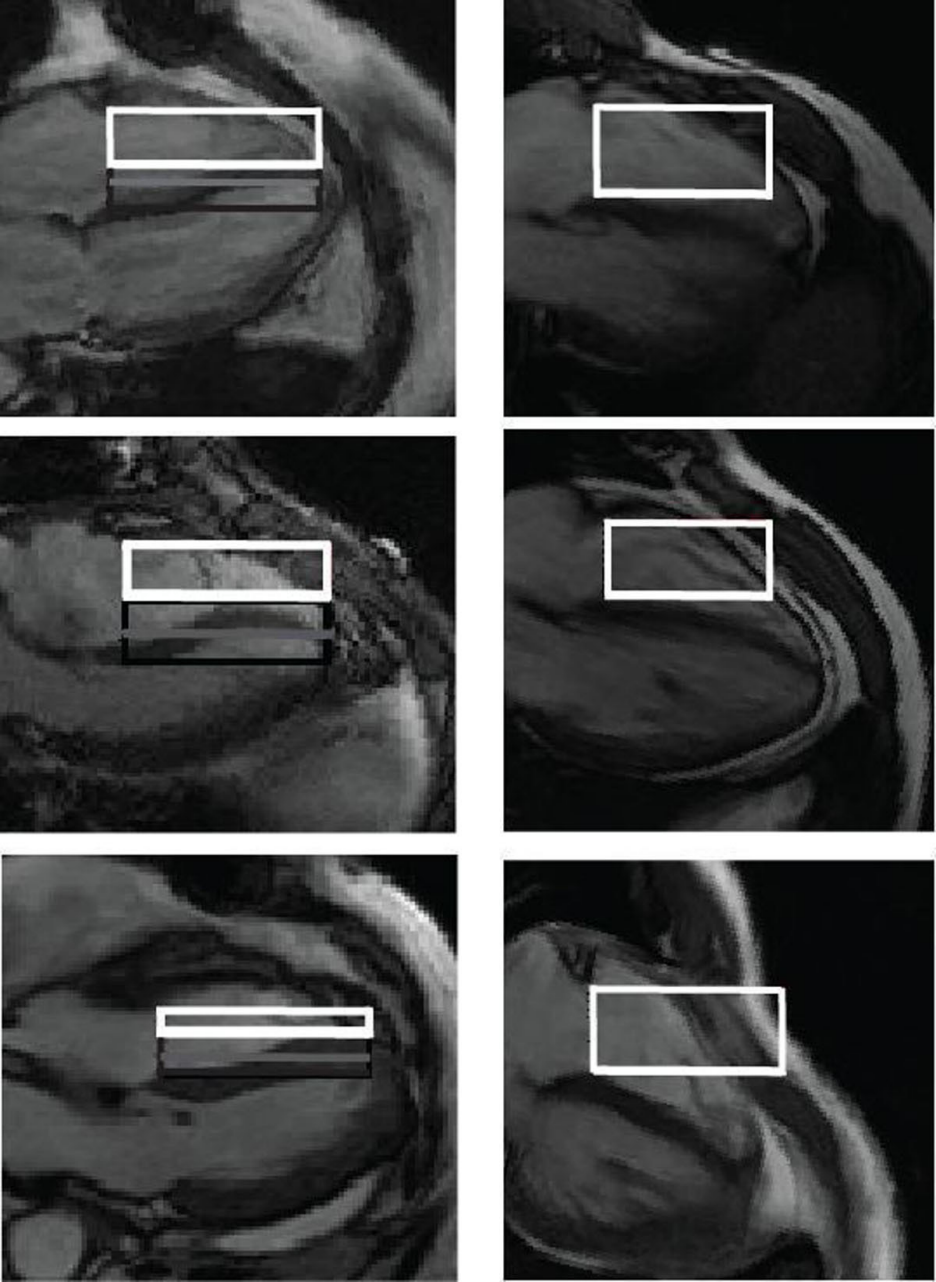


## Results

Septal bounce in CP is composed of two sequential movements, each consisting of an impingement of the septum from the RV towards the LV followed by recovery of this deformation. First movement, of larger amplitude, happens at 126 ± 57 ms following the opening of the mitral valve, while the second is noted at 39 ± 39 ms after the initiation of the atrial systole. The septal flow ratio at rapid ventricular filling (during first movement of the bounce) is 0.38 ± 0.19 in CP versus 0.01 ± 0.03 in controls, P < 0.0001, suggesting that the tricuspid blood inflow impacts the interventricular septum in CP and it does not in controls.

## Conclusions

The two movements of the septal bounce in CP patients correlate in time with the rapid diastolic filling and atrial systole and are possibly determined by cardiac mechanisms, i.e. the tricuspid blood inflow impacting the interventricular septum during diastole.

